# Evolution of Opinions on Social Networks in the Presence of Competing Committed Groups

**DOI:** 10.1371/journal.pone.0033215

**Published:** 2012-03-20

**Authors:** Jierui Xie, Jeffrey Emenheiser, Matthew Kirby, Sameet Sreenivasan, Boleslaw K. Szymanski, Gyorgy Korniss

**Affiliations:** 1 Department of Computer Science, Rensselaer Polytechnic Institute, Troy, New York, United States of America; 2 Department of Physics, Applied Physics, and Astronomy, Rensselaer Polytechnic Institute, Troy, New York, United States of America; 3 Social and Cognitive Networks Academic Research Center, Rensselaer Polytechnic Institute, Troy, New York, United States of America; Umeå University, Sweden

## Abstract

Public opinion is often affected by the presence of committed groups of individuals dedicated to competing points of view. Using a model of pairwise social influence, we study how the presence of such groups within social networks affects the outcome and the speed of evolution of the overall opinion on the network. Earlier work indicated that a single committed group within a dense social network can cause the entire network to quickly adopt the group's opinion (in times scaling logarithmically with the network size), so long as the committed group constitutes more than about 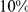 of the population (with the findings being qualitatively similar for sparse networks as well). Here we study the more general case of opinion evolution when two groups committed to distinct, competing opinions 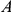 and 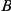, and constituting fractions 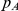 and 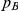 of the total population respectively, are present in the network. We show for stylized social networks (including Erdös-Rényi random graphs and Barabási-Albert scale-free networks) that the phase diagram of this system in parameter space 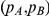 consists of two regions, one where two stable steady-states coexist, and the remaining where only a single stable steady-state exists. These two regions are separated by two fold-bifurcation (spinodal) lines which meet tangentially and terminate at a cusp (critical point). We provide further insights to the phase diagram and to the nature of the underlying phase transitions by investigating the model on infinite (mean-field limit), finite complete graphs and finite sparse networks. For the latter case, we also derive the scaling exponent associated with the exponential growth of switching times as a function of the distance from the critical point.

## Introduction

Since the seminal work of Gabriel Tarde [1] in the late 1800 s, the shaping of public opinion through interpersonal influence and conformity has been a subject of significant interest in sociology. This topic is especially relevant today due to the preponderance of online social media where individuals can influence and be influenced by their numerous and geographically scattered contacts. Public opinion on an issue is often shaped by the actions of groups that rigidly advocate competing points of view. The most evident example of such a process occurs during elections when multiple parties campaign to influence and win over the majority of voters. In this as well as other common scenarios, the predominant means of influencing public opinion involves some form of broadcast outreach such as television advertising, public demonstrations etc. However, even though factors exogenous to the network may have a significant effect on individuals becoming informed and engaged in particular issues [2], there is reason to believe that large scale changes in behavior or opinion are driven primarily through interpersonal influence events occurring within the network. Specifically in the context of rural campaigns, there is evidence that interpersonal channels constitute the dominant pathways for effecting individual behavior change, even when direct external influence is present [3]. Furthermore, with data on social networks becoming increasingly accessible, there has been a surge of interest in understanding how campaigns can be successfully won by leveraging pathways of social influence within the network, thus diminishing the need for, or complementing the effect of broadcast outreach.

Motivated by these observations, we study a simple model that enables us to draw useful insights on the evolution of opinions on a social network in the presence of two groups within the network that are committed to distinct, competing opinions on an issue. Within the limits of our model, one of the questions our work answers is the following. Suppose the majority of individuals on a social network subscribe to a particular opinion on a given issue, and additionally some fraction of this majority are unshakeable in their commitment to the opinion. Then, what should be the minimal fractional size of a competing committed group in order to effect a fast reversal in the majority opinion? In addition to answering this question quantitatively, we show the existence of two distinct types of phase transitions that can occur in the space of committed fraction pair values.

We model the dynamics of social influence using a two-opinion variant of the Naming Game [4]–[6] which also corresponds to a special case of the game introduced and studied in [7], [8]. The same model was referred to as the binary-agreement model in [9]. In this model, at any time, a node possesses either one of the two competing opinions (i.e. the node is in state 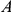 or state 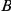), or both opinions simultaneously (state 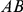). In a given time step, we choose a node randomly, designate it as the *speaker* and choose one of its neighbors randomly and designate it as the *listener*. The speaker proceeds to convey its opinion to the listener (chosen randomly if it possesses two) to the listener. If the listener possesses this opinion already, both speaker and listener retain it while eliminating all other opinions; otherwise, the listener adds the opinion to his list. A table of possible interactions and outcomes between node-pairs is provided in Table S1. We emphasize that each node interacts and is influenced only by its neighbors on the network. There is no element in our model that represents an external influence mechanism such as the use of media, public demonstrations, or door-to-door campaigns by members of the competing groups. Except for their being un-influencable, the committed nodes are assumed to be identical in all other respects to uncommitted nodes. In particular, committed nodes do not influence their neighbors at a different rate or with a higher strength than uncommitted nodes.

Opinion dynamics models involving committed individuals all subscribing to a unique opinion have been studied previously in [9]–[12]. The situation pertinent to this paper - that of two competing committed groups - has received considerably greater attention [11], [13]–[16]. Mobilia et al. [14] studied how the presence of zealots (equivalent to committed individuals) affected the eventual distribution of opinions (stationary magnetization) in the case of the voter model. They demonstrated that the distribution for a finite sized network was Gaussian, with a width inversely proportional to the square root of the number of zealots, and centered at 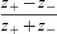 where 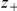, 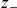, represent the fraction of zealots in the two competing states. Similarly to [14], Yildiz et al. [16] studied the properties of steady-state opinion distribution for the voter model with *stubborn* agents, but additionally considered the optimal placement of stubborn agents so as to maximally affect the steady-state opinion on the network. Interestingly, unlike in the model studied here, in the voter model, no transitions in steady-state magnetization are observed as the committed fraction pair values are smoothly varied. Biswas et al. [15] considered the effect of having rigid individuals in a one-dimensional system of binary opinion evolution, and demonstrated a power-law dependence for the decay of steady-state magnetization on the fraction of rigid individuals.The work done in [11], [13] is similar in spirit to our work here; however, an important difference is that these studies only considered the infinite-network size limit for complete graphs. We study finite networks, both complete and sparse, and provide semi-analytical arguments regarding timescales that become relevant when the network size is finite.

## Analysis

First, we study the mean-field version of the model, also being equivalent to the dynamics on the complete graph in the limit of infinite system size. We designate the densities of uncommitted agents in the states 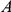, 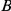 and 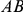 by 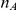, 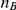 and 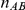. We also designate the fraction of nodes committed to state 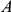, 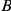 by 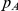, 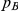 respectively. These quantities naturally obey the condition: 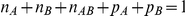. In the asymptotic limit of network size, and neglecting fluctuations and correlations, the system can be described by the following mean-field equations, for given values of the parameters 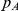 and 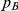:
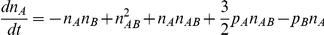


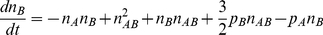
(1)The evolution of 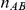 follows from the constraint on densities defined above. In general, the evolution of the system depends on the relative values of 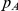 and 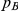. In the case of 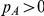, 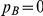 (or equivalently, 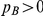, 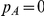) there is only a single group of committed nodes in the network, all of whom subscribe to the same opinion. This was the case studied in [9], [11], [12]. In this scenario, a transition is observed when this committed group constitutes a critical fraction of the total network. Specifically, the transition point separates two dynamical scenarios in the phase space, 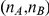, of uncommitted node densities. Below the critical value, the absorbing state (e.g., 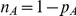, 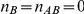 when 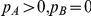) coexists in phase space with a stable mixed steady-state and an unstable fixed (“saddle”) point. At or above the critical value, the latter non-absorbing steady-state and the saddle point cease to exist. Consequently, for a finite system, reaching the (all 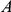) consensus state requires an exponentially long time when 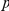 is less than the critical value. Beyond the critical value this time grows only logarithmically with network size. Note that this critical value or threshold is analogous to a spinodal point [17], [18] associated with an underlying first-order (or discontinuous) transition in equilibrium systems.

**Figure 1:**
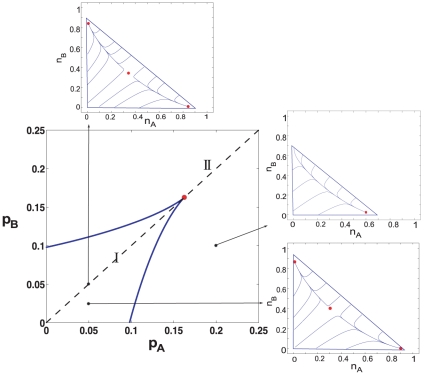
Mean-field picture in parameter space. The phase diagram obtained by integrating the mean-field Eqs. (1). The two lines indicate saddle-node bifurcation lines which form the boundary between two regions with markedly different behavior in phase space. For any values of parameters within the beak, denoted as region I, the system has two stable fixed points separated by a saddle point. Outside of the beak, in region II, the system has a single stable fixed point. The saddle-node bifurcation lines meet tangentially and terminate at a cusp bifurcation point.

In order to effectively characterize the behavior of the system governed by Eqs. (1) for 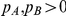, we systematically explore the parameter space 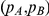 by dividing it into a grid with a resolution of 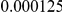 along each dimension. We then numerically integrate Eqs. (1) for each 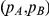 pair on this grid, assuming two distinct initial conditions, 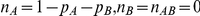 and 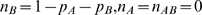, representing diagonally opposite extremes in phase space. The results of this procedure reveal the picture shown in Fig. 1 in different regions of parameter space. As is obvious, with non-zero values for both 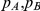, consensus on a single opinion can never be reached, and therefore all fixed points (steady-states) are non-absorbing. With 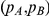 values within the region denoted as 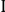 which we refer to as the “beak” (borrowing terminology used in [19]), the phase space contains two stable fixed points, separated by a saddle point, while outside the beak, in region 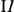, only a single stable fixed point exists in phase space. In region I, one fixed point corresponds to a state where opinion 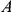 is the majority opinion (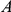-dominant) while the other fixed point corresponds to a state where opinion 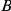 constitutes the majority opinion (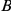-dominant). Figure 1 shows representative trajectories and fixed points in phase space, in different regions of parameter space. Similar phase diagrams have been found in other two-parameter systems in different contexts including chemical reactions [19] and genetic switches [20].

In order to study the nature of the transitions that occur when we cross the boundaries of the beak, we parametrize the system by denoting 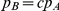 where 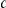 is a real number. Then, we systematically analyze the transitions occurring in two cases: (i) 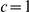 and (ii) 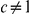. It can be shown that along the diagonal line 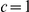 the system undergoes a cusp bifurcation at 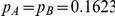. The movement of the fixed points as 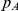 and 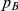 are smoothly varied along the diagonal line is shown in Figure S1. Henceforth, we denote the value of 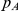 and 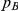 at the cusp as 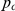. As is well known, at the cusp bifurcation two branches of a saddle-node (or fold) bifurcation meet tangentially [21]. These two bifurcation curves form the boundary of the beak shown in Fig. 1. A detailed analysis demonstrating that 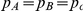 constitutes a cusp bifurcation, as well as a semi- analytical derivation of the bifurcation curves is provided in the Supporting Text S1 (Sections: 1, 2, 3). The cusp bifurcation point is analogous to a second-order (or continuous) critical point seen in equilibrium systems, while bifurcation curves are analogous to spinodal transition lines.

**Figure 2:**
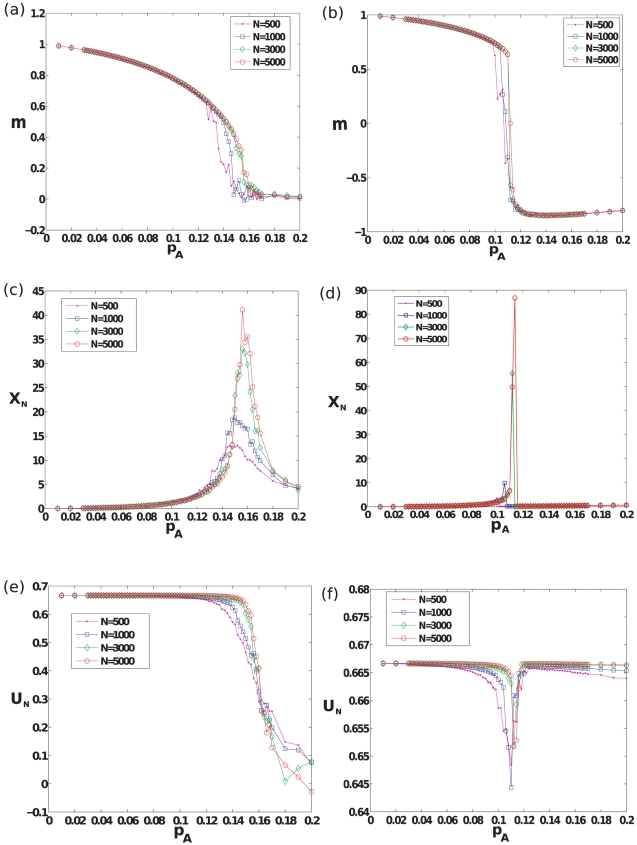
Behavior of typical order parameters as a function of linear trajectories of slope 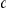 that pass through the origin, in parameter space for a complete graph. (a)–(b) Steady-state magnetization 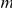 defined in the text, for successive 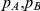 pairs along lines of slope 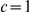 and 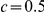 respectively that pass through the origin. The 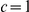 line in parameter space passes through the cusp point and gives rise to a second-order phase transition, while the 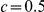 line passes through a point on the (right) bifurcation line giving rise to a first-order phase transition. Here 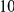 realizations of social influence dynamics were performed for each 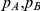 pair, starting from the initial condition 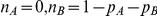, and the magnetization was measured conditioned on the system remaining in the steady state that it initially converged to. (c)–(d) Binder cumulant 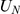 defined in the text for successive 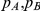 pairs along lines of slope 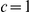 and 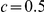 respectively, that pass through the origin. (e)–(f) Scaled variance, 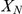, defined in the text for successive 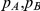 pairs along lines of slope 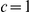 and 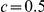 respectively, that pass through the origin. Data for (c),(d),(e) and (f) were generated from 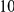 realizations of the social influence dynamics, per 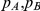 pair, for each of two initial conditions: 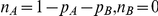 and 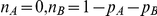.

Next, we study the stochastic evolution of opinions on finite-sized complete graphs through simulations. Here, we systematically vary 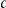 from 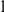 to 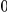 to obtain the right bifurcation curve, and therefore by virtue of the 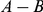 symmetry in the system, also obtain the left bifurcation curve. In particular for a given value of 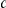 we obtain the transition point by varying 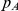 (with 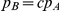) and measuring the quantity:
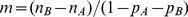
(2)which we utilize as an order parameter. The above order parameter is analogous to the “magnetization” in a spin system as it captures the degree of dominance of opinion 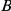 over opinion 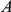 and is conventionally used to characterize the nature of phase transitions exhibited by such a system (see Figs. 2(a),(b)).

Another quantity, the Binder cumulant, defined as
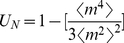
(3)for a system of size 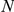, is commonly used to distinguish between different types of phase transitions [17]. The utility of the Binder cumulant comes from the markedly different signatures we expect it to produce along a spinodal trajectory (e.g. 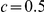) - one that passes through the spinodal line - and one along a trajectory that passes through the critical point (e.g., along the diagonal, 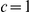). This difference arises from the following distinction in the evolution of the distribution of 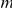, 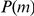, along these trajectories. Along a spinodal trajectory starting from a point where 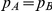, an initially symmetric (about 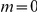), bimodal 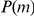 becomes asymmetric and unimodal upon crossing the spinodal line, with the single mode eventually becoming a delta function. In contrast, along the diagonal trajectory in parameter space, 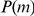 is initially a double-delta distribution (for 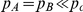), symmetric about 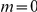, and it smoothly transitions to a zero-centred gaussian distribution as the critical point is crossed. The definition of 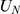 indicates that 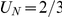 for a delta function distribution (also for a symmetric, double-delta distribution about 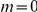), while 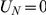 for a zero-centered Gaussian distribution, and thus readily yields the limiting 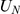 values at both extremes of the spinodal and diagonal trajectory. As illustrated in Figs. 2(c) and (d), 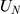 as a function of 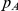 shows distinct behaviors for 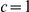 and 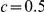, indicating the existence of a second-order (or continuous) transition point at 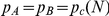 (Fig. 2(c)) and first-order (or discontinuous) phase transition points (Fig. 2(d)) along off-diagonal trajectories [17], respectively. The second-order critical point 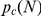 converges to the mean-field value, 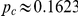, as 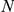 becomes larger. The dip observed in 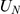 along the off-diagonal trajectory serves as an excellent estimator of the location of the first-order (spinodal) transition for a finite network. Thus, to reiterate, for a finite network, the second-order transition point and the first-order transition (spinodal) lines are respective analogues of the cusp bifurcation point and the saddle-node bifurcation curves observed in the mean-field case.

The fluctuations of the quantity 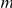 can also be used to identify a transition point, particularly for the case of the second-order transition. In particular, in formal analogy with methods employed in the study of equilibrium spin systems, the scaled variance:
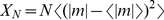
(4)serves as an excellent estimate for the second-order transition point 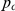 for a finite network. As shown in Fig. 2(e), 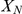 peaks at a particular value of 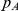, with the size of the peak growing with 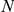 (and expected to diverge as 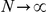). In the case of the spinodal transition, one studies fluctuations of 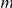 (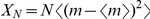) restricted to the metastable state [22], [23] until the spinodal point (Fig. 2(f)) at which the metastable state disappears, and fluctuations of 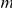 in the unique stable state beyond the spinodal point (Fig. 2(f)).

**Figure 3:**
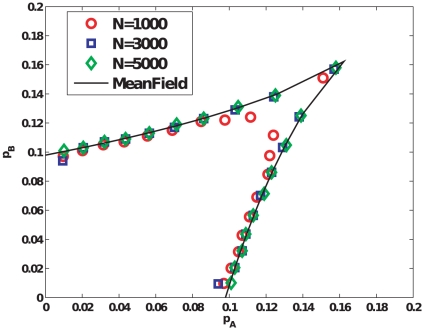
Picture in parameter space for a complete graph obtained from analytical and simulation results. The bifurcation lines and the cusp point in parameter space were obtained analytically from the mean field equations and are compared with those found using simulations for finite-sized complete graphs. Analytical and simulation curves show excellent agreement as 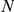 increases. The location of the transition occurring across the bifurcation curve was obtained using the Binder cumulant 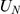 (Fig. 2(d)), while the location of the cusp point was obtained by using variance of 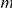 (Fig. 2(e)). For both analytical and simulation results, the bifurcation curves are obtained by identifying the critical points that lie on linear trajectories in parameter size described by 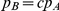. This process is carried out for different values of 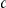 between 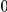 and 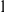 at intervals of 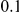, and for each value of 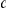, 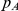 is varied at a resolution of 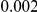. In simulations, for each such combination of 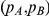 obtained, we perform averages over 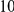 realizations of the social influence dynamics, for each of two initial conditions, 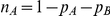, and 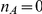, with 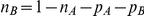 for each case.

Figure 3 shows the bifurcation (spinodal) lines obtained via simulations of finite complete graphs by using the Binder cumulant (Fig. 2(d))to identify the location of the spinodal phase transition, and demonstrates that its agreement with the mean-field curves improves as 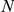 grows. The cusp points shown here are identified in simulations as the locations where 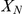 reaches its peak value (Fig. 2(e)).

**Figure 4:**
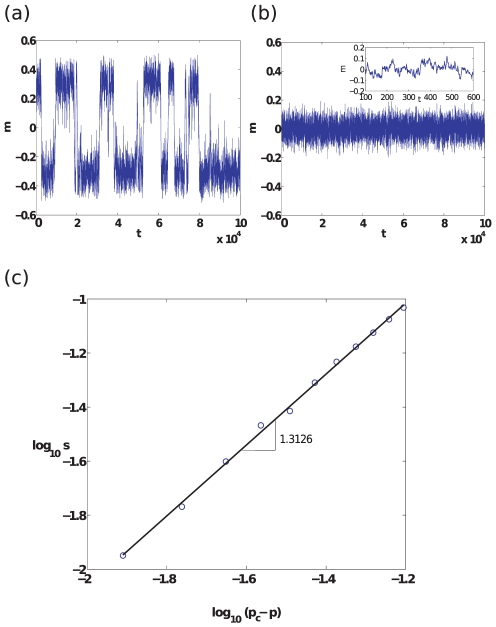
Evolution of order parameter 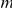 and the exponential growth in switching time as a function of distance from the second-order critical point. (a) Switches in the value of 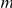 as a function of time 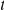 for a sample evolution (with initial transient removed) of the system when 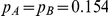 (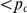). This reflects the system repeatedly switching between the 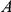-dominant steady-state (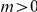) and the 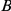-dominant steady-state (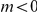). (b) Sample evolution of the system (with initial transient removed) for 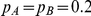 (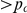). The system fluctuates randomly about the only existing steady-state in which densities of 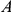 and 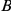 nodes are equal. (c) The dependence of 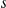 in the exponential scaling 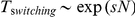 when 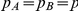 (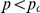) as a function of 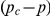, obtained using the eikonal approximation (see SI: Section 4).

In the region within the beak, the switching time between the co-existing steady-states represents the longest time-scale of relevance in the system. The switching time is defined as the time the system takes to escape to a distinct co-existing steady-state, after having been trapped in one of the steady-states. Figures 4(a) and (b) show sample evolutions of the system, demonstrating respectively, the switching between steady-states within the beak, and the fluctuations about the single steady state outside the beak. In stochastic systems exhibiting multistability or metastability, it is well known that switching times increase exponentially with 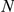 for large 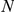 (the weak-noise limit) [19], [24]–[26] Furthermore, the exponential growth rate of the switching time in such cases can be determined using the eikonal approximation [19], [27]. The basic idea in the approximation involves (i) assuming an eikonal form for the probability of occupying a state far from the steady-state and (ii) smoothness of transition probabilities in the master equation of the system. This allows the interpretation of fluctuational trajectories as paths conforming to an auxilliary Hamilton-Jacobi system. This in turn enables us to calculate the probability of escape allowing an *optimal fluctuational path* that takes the system from the vicinity of the steady-state to the vicinity of the saddle point of the deterministic system. The switching time is simply the inverse of the probability of escape along this optimal fluctuational path. We defer details of this procedure to Supporting Text S1: Section 4. Using this approach we find that for the symmetric case, 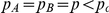, the exponential growth rate of the switching time 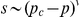 with 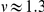 (Fig. 4 (c) ). Thus, along the portion of the diagonal within the beak:
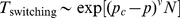
(5)Outside the beak, the time to get arbitrarily close to the sole steady-state value grows logarithmically with 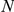 (not shown).

The results presented so far show that there exists a transition in the time needed by a committed minority to influence the entire population to adopt its opinion, even in the presence of a committed opposition (i.e. in the case where both 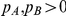), as long as 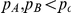. (Note that the case 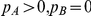 was considered in [9]). For example, assume that initially all the uncommitted nodes adopt opinion 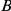, and that 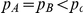. Then, the steady-state that the system reaches in 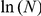 time is the one in which the majority of nodes hold opinion 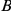. Despite the fact that there exist committed agents in state 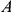 continuously proselytizing their state, it takes an exponentially long time before a large (spontaneous) fluctuation switches the system to the 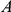-dominant steady-state. For identical initial conditions, the picture is qualitatively the same if we increase 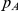 keeping 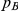 fixed, as long as 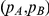 lies within the beak. However, when 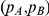 lies on the bifurcation curve or beyond, the 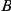-dominant steady-state vanishes, and with the same initial conditions - where 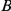 is the initial majority - it takes the system only 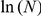 time to reach the 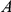-dominant state (the only existing steady-state). Thus, for every value of an existing committed fraction 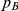 (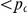) of 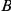 nodes, there exists a corresponding critical fraction of 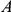 nodes beyond which it is guaranteed that the system will reach an 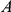 dominant state in 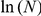 time, irrespective of the initial conditions. However, for any trajectory in the parameter space in a region where either 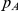 or 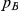 is (or both are) greater than 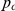, no abrupt changes in dominance or consensus times are observed. Instead, the dominance of 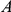 or 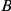 at the single fixed point smoothly varies as the associated committed fractions are varied. Moreover, the system always reaches this single fixed point in 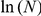 time.

**Figure 5:**
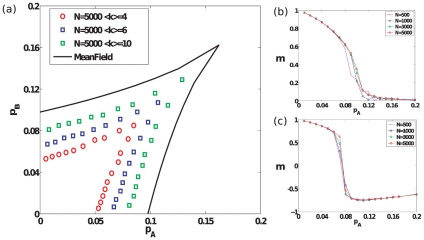
Results for Erdös-Rényi random graphs. (a) The bifurcation lines and cusp point in parameter space obtained through simulations of Erdös-Rényi random graphs of size 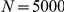 with different average degrees. The mean-field analytical curve is shown for comparison. For simulation results, the bifurcation curves are obtained by identifying the critical points that lie on linear trajectories described by 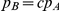 in parameter space. This process is carried out for different values of 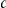 between 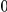 and 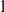 at intervals of 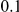, and for each value of 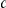, 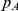 is varied at a resolution of 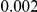. For each such combination of 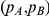 obtained, we perform averages for quantities of interest over 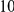 realizations of networks (with a single realization of the social influence dynamics per network), for each of two initial conditions, 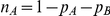 and 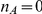 with 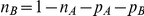 in each case. (b)–(c) Steady-state magnetization for ER graphs with 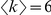 and different sizes 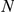, as parameter pair values are varied successively along slope 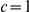 and slope 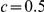 lines in parameter space respectively.

**Figure 6:**
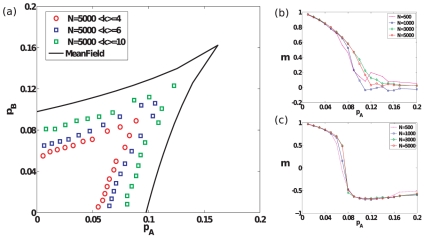
Results for Barabási-Albert networks. (a) The bifurcation lines and cusp point in parameter space obtained through simulations of Barabási-Albert networks of size 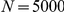 with different average degrees. For simulation results, the bifurcation curves are obtained by a similar method as described in the legend of Fig. 5(a). (b)–(c) Steady-state magnetization for BA networks with 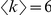 and different sizes 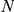, as parameter pair values are varied successively along slope 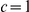 and slope 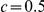 lines in parameter space respectively.

**Figure 7:**
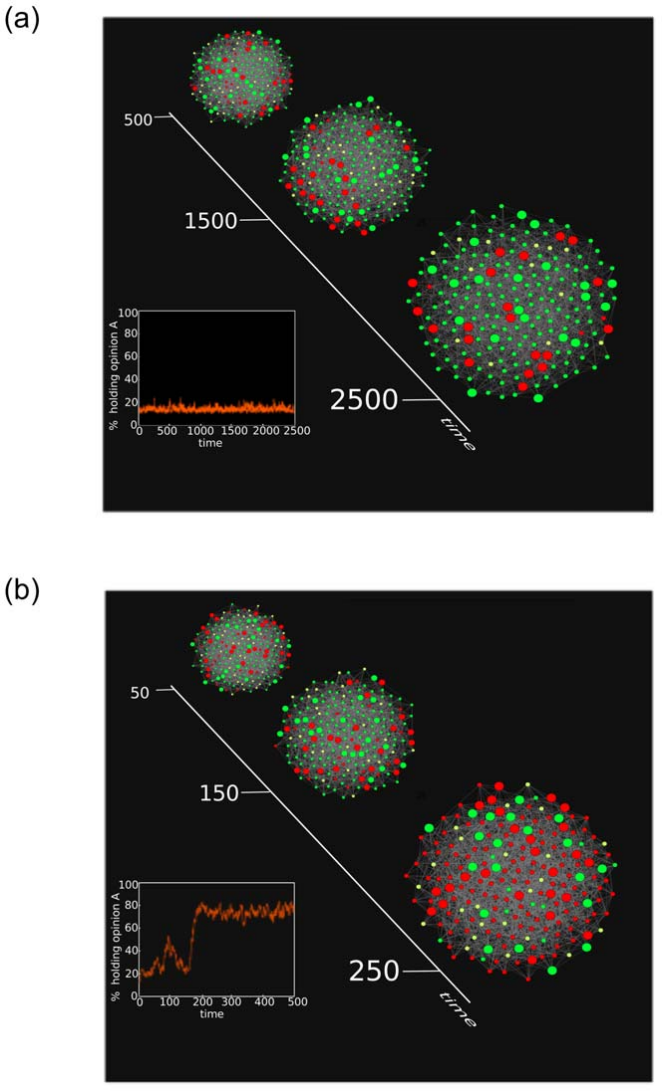
Visualization of opinion evolutions. The evolution of opinions on an ER random graph with 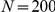 and 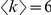 for two 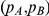 pairs. In each case 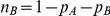 and 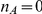. Nodes holding opinion 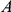 are depicted in red, while nodes holding opinion 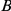 are shown in green. Nodes with larger diameters are committed nodes. Top: The case 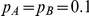 for which the system is in region I in parameter space (following the terminology of Fig. 1, and the system is trapped in a 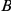-dominant steady-state. Even after 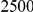 time steps, the system continues to remain trapped in this state (inset) with 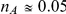. Bottom: The case 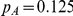 and 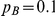 for which the system is in the region II, and undergoes an abrupt transition (inset) to the 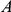-dominant state within 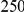 time steps.

Finally, we study how opinions evolve in the presence of committed groups on sparse graphs, most relevant to social networks. We study Erdös-Rényi (ER) random graphs [28] as well as Barabási-Albert networks [29]. For each of these sparse networks, we find the same qualitative behavior as found for the complete graph. As shown in Figs. 5, 6, as the average degree of the sparse networks increases, the bifurcation lines in parameter space tend to approach their mean-field counterparts. Although we do not study sparse networks analytically here, we note that in another instance of a phase transition for a similar model studied in [7], it was demonstrated using heterogeneous mean-field equations that the behavior of sparse networks is qualitatively similar to that of complete graphs. Figure 7 visually depicts typical instances of the evolution of opinions on an ER random graph for 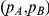 values within and outside the beak.

## Discussion

Using a simple model, we have explored and quantified possible outcomes for the evolution of opinions on a social network in the presence of groups committed to competing opinions. Broadly speaking, our results indicate that as long as the fraction, 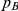, of nodes committed to a given opinion 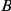 is held fixed at a value less than a critical value 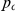, it is possible to induce the network to quickly tip over to a state where it widely adopts a competing opinion 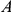, by introducing a fraction of nodes committed to opinion 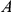. The value of the competing committed fraction, 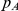, at which this tipping point arises depends on the value of 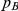, and is determined by the bifurcation curve (see Fig. 1). Importantly, for a given value of 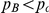, the excess commitment 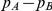 required for the network to tip over to 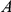 is a decreasing function of 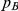 that reaches zero when 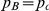. While the critical value 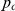 itself may depend on the network structure and its size, the feature described above holds for the three different classes of networks studied here. A corollary to this feature is that if the committed fraction 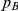 is held fixed at a value greater than pc, increasing the competing committed fraction 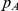 only yields continuous incremental gains in the adoption of A (i.e., no tipping point or discontinuous changes in opinions exist). We analytically determine that 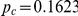 for infinite-sized complete graphs, which as observed from our simulation results in Figs. 5, 6 appears to constitute a good upper bound to the value of 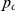 for sparse networks.

Our results could be of utility in situations where public opinion is deadlocked due to the influence of competing committed groups. Perhaps one example of such a situation is the observed lack of consensus in the U.S. on the existence of human-induced climate change. Indeed, there is evidence in this particular case that the commitment of individuals to particular political ideologies may have an effect on their opinions [30].

Another scenario to which our model could bear some relevance is the adoption of competing industrial standards. Particularly in situations where a network of entities collaborate or are interdependent, there is a natural attempt at agreement in standards or protocols between interacting members. A classic example of this scenario is the case of the Sellers' screw manufacturing standard that proliferated despite competition from the Whitworth standard [31]. A key factor responsible for the eventual success of the Sellers standard was William Sellers' leveraging of his connections to corporations and manufacturers [32], whom he persuaded to become adopters of his standard. Furthermore, the network of interdependencies between industries at the dawn of the mass-manufacturing era played an important role in the adoption of the standard becoming widespread. It should be pointed out that in this case, the uncommitted members of the population initially adhered to neither standard - this situation can however be accommodated in our model by assigning each uncommitted his own unique “opinion” to begin with in close analogy to initial conditions for the original Naming Game [4], [5].

A more recent example of such a scenario is the competition between Flash and HTML5 in web-development. There is speculation that Flash, which until recently was the predominant platform for animated web content, is gradually ceding its dominance to HTML5 as a result of the increasing market-share of Apple's mobile devices which exclusively support the latter [33].

A potential competition between DC fast charging standards is also expected as electric vehicles become increasingly popular with consumers. The front-runners in the mass manufacture of electric vehicles have opted for the CHAdeMO standard, and charging stations compatible with the standard have begun proliferating in the US, Europe and Japan [34], [35]. An alternative to CHAdeMO currently being developed by the Society for Automotive Engineers (SAE), which governs the development of standards in the US automotive industry, is being touted by some car manufacturers as more cost effective as well as technologically superior. However, by the time the first cars employing the SAE standard hit the market, CHAdeMO charging stations are expected to be rather widespread, thus making a competition between the two inevitable [34], [36]. As new collaborations are forged between car-makers especially in the area of electric vehicle development [37], [38] (in addition to collaborations with energy suppliers), the outcome of this competition could be significantly influenced by manufacturers who are already committed to one of the two standards through their investment in them.

To conclude, we have presented results from a simple, abstract model for understanding how opinions on a social network evolve through social influence when there are multiple groups within the network dedicated to competing opinions. Despite the simplicity of our model, we believe the insights provided here form a useful theoretical complement to data-driven studies [39] and randomized evaluations [40] aimed at understanding the spread of opinions.

**Note:** Subsequent to this paper's initial posting on arxiv.org on 12/31/2011 and its acceptance for publication with minor revisions on 1/26/2012, S. Jolad sent us independent unpublished results by D. Linford, P. Hochendoner, A. Reagan, and S. Jolad addressing competing committed groups on the complete graph.

## Supporting Information

Figure S1
**Movement of fixed points as** 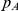 **and** 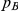 **are smoothly varied along the diagonal line** 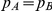 **.** For 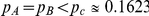 three fixed points exist, two of which are stable, and the third is unstable. For 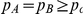, only a single stable fixed point exists.(EPS)

Table S1
**Possible interactions and respective outcomes in the model of social influence - a two-opinion variant of the Naming Game - that we study.** Nodes can possess opinion 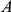, 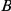 or 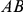, and opinion updates occur through repeated selection of speaker-listener pairs. Shown in the left column are the opinions of the speaker (first) and listener (second) before the interaction, and the opinion voiced by the speaker during the interaction is shown above the arrow. The column on right shows the states of the speaker-listener pair after the interaction.(PDF)

Supporting Text S1 **Mean-field analysis for bifurcations in parameter space and scaling of switching time with system size.**
(PDF)
