# Supplementary material for: Evolution of Opinions on Social Networks in the Presence of Competing Committed Groups
Source: PLoS One. 2012 Mar 20;7(3):e33215. doi: 10.1371/journal.pone.0033215 (PMC3308977; doi:10.1371/journal.pone.0033215)
Supplement: Table S1 — Possible interactions and respective outcomes in the model of social influence - a two-opinion variant of the Naming Game - that we study. Nodes can possess opinion , or , and opinion updates occur through repeated selection of speaker-listener pairs. Shown in the left column are the opinions of the speaker (first) and listener (second) before the interaction, and the opinion voiced by the speaker during the interaction is shown above the arrow. The column on right shows the states of the speaker-listener pair after the interaction. (PDF) [file pone.0033215.s002.pdf]

Before interaction	After interaction
A $\overset{A}{\rightarrow}$ A	A - A
A $\overset{A}{\rightarrow}$ B	A - AB
A $\overset{A}{\rightarrow}$ AB	A - A
B $\overset{B}{\rightarrow}$ A	B - AB
B $\overset{B}{\rightarrow}$ B	B - B
B $\overset{B}{\rightarrow}$ AB	B - B
AB $\overset{A}{\rightarrow}$ A	A - A
AB $\overset{A}{\rightarrow}$ B	AB - AB
AB $\overset{A}{\rightarrow}$ AB	A - A
AB $\overset{B}{\rightarrow}$ A	AB - AB
AB $\overset{B}{\rightarrow}$ B	B - B
AB $\overset{B}{\rightarrow}$ AB	B - B

Table S1: Possible interactions and respective outcomes in the model of social influence - a two-opinion variant of the Naming Game - that we study. Nodes can possess opinion A, B or AB, and opinion updates occur through repeated selection of speaker-listener pairs. Shown in the left column are the opinions of the speaker (first) and listener (second) before the interaction, and the opinion voiced by the speaker during the interaction is shown above the arrow. The column on right shows the states of the speaker-listener pair after the interaction.
