# Supplementary material for: Evolution of Opinions on Social Networks in the Presence of Competing Committed Groups
Source: PLoS One. 2012 Mar 20;7(3):e33215. doi: 10.1371/journal.pone.0033215 (PMC3308977; doi:10.1371/journal.pone.0033215)
Supplement: Supporting Text S1 — Mean-field analysis for bifurcations in parameter space and scaling of switching time with system size. (PDF) [file pone.0033215.s003.pdf]

Supporting Text S1
Evolution of opinions on social networks in the presence of competing committed groups

Jierui Xie, Jeffrey Emenheiser, Matthew Kirby,
Sameet Sreenivasan, Boleslaw K. Szymanski and Gyorgy Korniss

Contents

1 Analysis of steady states for $p_A = p_B$: existence of a critical value $p_A = p_B = p_c$. **2**

2 Existence of a cusp point **3**

3 Mapping out the bifurcation curves (first order transition lines) **4**

4 Optimal fluctuational paths, the eikonal approximation and switching times between co-existing stable states **5**

1 Analysis of steady states for $p_A = p_B$: existence of a critical value $p_A = p_B = p_c$.

For notational simplicity we replace n_A by x and n_B by y. The mean field equations describing the system with $p_A = p_B = p$, $0 < p \leq 0.5$ then are:

$$\begin{aligned} \frac{dx}{dt} &= -xy + (1-x-y-2p)^2 + x(1-x-y-2p) + \frac{3}{2}p(1-x-y-2p) - px \\ \frac{dx}{dt} &= -xy + (1-x-y-2p)^2 + y(1-x-y-2p) + \frac{3}{2}p(1-x-y-2p) - py \end{aligned} \tag{1}$$

where $n_{AB} = 1 - x - y - 2p$. In the steady state, $dx/dt = dy/dt = 0$, and the resulting equations can be solved to yield four solutions for (x, y). Out of these one solution lies outside the valid range for all feasible values of p, i.e., $0 < p \leq 0.5$. The valid fixed points for Eqs. 1 are:

$$\begin{aligned} x_1 &= \frac{3}{2} - \frac{1}{2}\sqrt{5-2p} - p \\ y_1 &= \frac{3}{2} - \frac{1}{2}\sqrt{5-2p} - p \end{aligned}$$

$$\begin{aligned} x_2 &= \frac{1}{2} + \frac{1}{2}\sqrt{1-p^2-6p} - \frac{3}{2}p \\ y_2 &= \frac{1}{2} - \frac{1}{2}\sqrt{1-p^2-6p} - \frac{3}{2}p \end{aligned}$$

$$\begin{aligned} x_3 &= \frac{1}{2} - \frac{1}{2}\sqrt{1-p^2-6p} - \frac{3}{2}p \\ y_3 &= \frac{1}{2} + \frac{1}{2}\sqrt{1-p^2-6p} - \frac{3}{2}p \end{aligned}$$

Since the solutions are symmetric in x and y, in order to investigate the range of p over which these solutions are valid, we restrict our analysis to y. The solution y_1 is valid for all values of p. For y_2, y_3 to be valid solutions, we require $U(p) = 1 - p^2 - 6p \geq 0$. $U(p)$ is a monotonically decreasing function for $p > 0$, and the value of p at which $U(p)$ first crosses zero is the critical point.

$$p_c = \sqrt{10} - 3 \approxeq 0.1623. \tag{2}$$

Thus, there exist three fixed points in the range $[0, p_c]$. In the range $(p_c, 0.5]$ only one valid fixed point exists, viz. (x_1, y_1).

We can further examine the stability of the obtained fixed points. Linear stability analysis yields the following stability matrix:

$$Q = \begin{bmatrix} -1 - \frac{p}{2} & -2 + 2y^* + \frac{5}{2}p \\ -2 + 2x^* + \frac{5}{2}p & -1 - \frac{p}{2} \end{bmatrix} \tag{3}$$

where (x^*, y^*) is the fixed point under consideration.

The eigenvalues of the stability matrix at the fixed point are given by $\lambda = -(2+p) \pm \sqrt{26p^2 + (20(x^* + y^*) - 36) + 16(1 - x^* - y^* + x^* y^*)}$, and examination of the real part of these eigenvalues indicates that (x_2, y_2) and (x_3, y_3) are stable fixed points, and (x_1, y_1) is an unstable fixed point (saddle point) for $p \leq p_c = 0.1623$. For $p > p_c$, (x_1, y_1), the only valid fixed point, is a stable fixed point. Figure S1 shows the movement of the fixed points in the phase space as a function of p.

2 Existence of a cusp point

Suppose that a *one*-dimensional parameter(α) dependent system

$$\frac{dx}{dt} = f(x; \alpha), x \in \Re^1, \alpha \in \Re^m \tag{4}$$

with smooth function f, has an equilibrium at $x = 0$ for $\alpha = 0$, and let $f_x(0;0) = 0$ and $f_{xx}(0;0) = 0$ hold. Further, assume that the non-degeneracy conditions (e.g., $f_{xxx}(0;0) \neq 0$) are satisfied. Then the system undergoes a *cusp* bifurcation at $x = 0$ [1].

We prove that such a cusp bifurcation is encountered in our system (i.e., Eq. 1) at $p_A = p_B = p_c$ as we move along the diagonal in parameter space ($p_A = p_B$). Note that our system is two-dimensional. To be able to use the above theory, we first need to reduce the dimensionality of our system. The Center Manifold Theorem [2] guarantees the existence of a one-dimensional center manifold to which we can restrict our system, and such a system preserves the same behavior as the original system in the vicinity of the steady-state under consideration. Once we get the restricted system, we can perform the usual bifurcation analysis in one-dimensional system. Following this idea, we first shift the coordinates such that the origin is located at the critical point we found from the $p_B = p_A$ case (for simplicity, we denote p_A by p and p_B by r), i.e., $(x_0, y_0; p_0, r_0) = (0.2565, 0.2565; \sqrt{10} - 3, \sqrt{10} - 3)$. In the shifted coordinates, the eigenvalues and eigenvectors are given by $\Lambda = [0; -2.1623]$ and $V = [-0.7071, 0.7071; 0.7071, 0.7071]$. Using transformation $[\tilde{x}\ \tilde{y}]^T = V[x\ y]^T$ and after some algebraic manipulations, we obtain in the new co-ordinate system:

$$\begin{aligned}
\frac{d\tilde{x}}{dt} &= 0.7071(1.5p + 0.2434)(p + r + 1.414\tilde{y} - 0.1623) \\
&\quad -0.7071(1.5r + 0.2434)(p + r + 1.414\tilde{y} - 0.1623) \\
&\quad -0.7071(0.7071\tilde{x} + 0.7071\tilde{y} + 0.2566)(p + r + 1.414\tilde{y} - 0.1623) \\
&\quad -0.7071(p + 0.1623)(0.7071\tilde{x} + 0.7071\tilde{y} + 0.2566) \\
&\quad +0.7071(0.7071\tilde{y} - 0.7071\tilde{x} + 0.2566)(p + r + 1.414\tilde{y} - 0.1623) \\
&\quad +0.7071(r + 0.1623)(0.7071\tilde{y} - 0.7071\tilde{x} + 0.2566) \\
\frac{d\tilde{y}}{dt} &= -0.7071(1.5r + 0.2434)(p + r + 1.414\tilde{y} - 0.1623) \\
&\quad -0.7071(1.5p + 0.2434)(p + r + 1.414\tilde{y} - 0.1623) \\
&\quad -0.7071(0.7071\tilde{x} + 0.7071\tilde{y} + 0.2566)(p + r + 1.414\tilde{y} - 0.1623) \\
&\quad -0.7071(p + 0.1623)(0.7071\tilde{x} + 0.7071\tilde{y} + 0.2566) \\
&\quad -0.7071(0.7071\tilde{y} - 0.7071\tilde{x} + 0.2566)(p + r + 1.414\tilde{y} - 0.1623) \\
&\quad -1.414(0.7071\tilde{x} + 0.7071\tilde{y} + 0.2566)(0.7071\tilde{y} - 0.7071\tilde{x} + 0.2566) \\
&\quad -0.7071(r + 0.1623)(0.7071\tilde{y} - 0.7071\tilde{x} + 0.2566) \\
&\quad +1.414(p + r + 1.414\tilde{y} - 0.1623)^2
\end{aligned} \tag{5}$$

Next, we use a quadratic approximation for the center manifold of the above system [2] i.e. we assume $\tilde{y} = h(\tilde{x}) = \frac{1}{2} w \tilde{x}^2$. We can find w by comparing two expressions obtained for $\frac{d\tilde{y}}{dt}$; the

first is obtained by using $\frac{d\tilde{y}}{dt} = \frac{d\tilde{y}}{d\tilde{x}}\frac{d\tilde{x}}{dt}$ and then using the first equation in Eq. 5 and the quadratic approximation for $\tilde{y}$; the second is obtained by direct substitution of the quadratic approximation into the second equation in Eq. 5. Doing this yields:

$$\tilde{y} = h(\tilde{x}) = -0.7071\tilde{x}^2/(4p + 4r - 2.1620)$$

Hence we obtain the following one dimensional system restricted to the one-dimensional center manifold:

$$\begin{aligned}
\frac{\partial \tilde{x}}{\partial t} \quad = \quad & 0.1814r - 0.1814p - \tilde{x}(1.5p + 1.5r) \\
& +0.7071(1.5p + 0.2434)(p + r - 0.1623) \\
& -0.7071(1.5r + 0.2434)(p + r - 0.1623) \\
& -\tilde{x}^2(0.7071(1.5p + 0.2434)/(4p + 4r - 2.1623) \\
& -0.7071(1.5r + 0.2434)/(4p + 4r - 2.1623) \\
& -0.7071(p + 0.1623)/(8p + 8r - 4.3246) \\
& +0.7071(r + 0.1623)/(8p + 8r - 4.3246)) \\
& +\tilde{x}^3/(4p + 4r - 2.1623)
\end{aligned} \tag{6}$$

It is easy to check that the origin in this transformed system satisfies the necessary conditions for a cusp bifurcation. The origin of this transformed system corresponds to the point $p_A = p_B = p_c$ in our original system Eq. 1. Thus, the system undergoes a cusp bifurcation at $p_A = p_B = p_c$ where $p_c = \sqrt{10} - 3 \approx 0.1623$.

3 Mapping out the bifurcation curves (first order transition lines)

In order to map out the first-order transition line (bifurcation curve) we adopt a semi-analytical approach. We assume $p_B = cp_A$ with $c < 1$ to obtain the lower bifurcation curve (symmetry of the system allows us to obtain the upper bifurcation curve, given the lower one). Using Eqs. 1, the fixed point condition becomes (for simplicity, we denote p_A by p):

$$\begin{aligned}
f(x, y, p) \quad \equiv \quad & -xy + (1 - x - y - (1 + c)p)^2 + x(1 - x - y - (1 + c)p) \\
+ \quad & \frac{3}{2}p(1 - x - y - (1 + c)p) - cpx = 0 \\
g(x, y, p) \quad \equiv \quad & -xy + (1 - x - y - (1 + c)p)^2 + y(1 - x - y - (1 + c)p) \\
+ \quad & \frac{3}{2}cp(1 - x - y - (1 + c)p) - py = 0
\end{aligned}$$

In addition, for a fold bifurcation, we also require that the stability matrix has an eigenvalue with zero real part. Since, the valid solutions in our case are always real, this is equivalent to requiring the determinant of the stability matrix to be zero. Thus the condition $|Q| = 0$ (with Q given by Eq. 3) along with Eqs. 7 enable us to determine for a given c, the location (p_A, cp_A) at which the bifurcation occurs. By numerically solving these equations for different values of c, $0 < c \leq 1$ at intervals of 0.1, we obtain the lower bifurcation curve shown in Fig. 2 of the main text.

4 Optimal fluctuational paths, the eikonal approximation and switching times between co-existing stable states

The master equation for our system takes the general form:

$$\frac{\partial P(\mathbf{X},t)}{\partial t} = \sum_{\mathbf{r}} \Big[W(\mathbf{X}-\mathbf{r},\mathbf{r})P(\mathbf{X}-\mathbf{r},t) - W(\mathbf{X},\mathbf{r})P(\mathbf{X},t) \Big]$$

where $\mathbf{X} = [N_A \ N_B]^T$ denotes the (macro) state of the system as vector whose elements are the numbers of uncommitted nodes in state A and B respectively, $W(\mathbf{X},\mathbf{r})$ is the probability of the transition from $\mathbf{X}$ to $\mathbf{X}+\mathbf{r}$, and $\mathbf{r}$ runs over the allowed set of displacement vectors in the space of macro-states. For our system, $\mathbf{r}$ runs over $[1 \ 0]^T, [0 \ 1]^T, [2 \ 0]^T, [0 \ 2]^T, [-1 \ 0]^T, [0 \ -1]^T$. The deterministic equations can be derived from this master equation and yield:

$$\frac{d\mathbf{X}_{\text{det}}}{dt} = \sum_{\mathbf{r}} \mathbf{r} W(\mathbf{X}_{\text{det}},\mathbf{r})$$

The Wentzell-Friedlin theory [5, 6] assumes that for any path $[\mathbf{X}]$ in configuration space:

$$\mathcal{P}([\mathbf{X}]) \sim \exp(-\mathcal{S}([\mathbf{X}]))$$

with $\mathcal{S}([\mathbf{X}^*]) = 0$ for the deterministic path $[\mathbf{X}^*]$. It follows that the dominant contribution to the probability of a fluctuation that brings the system to state $\mathbf{X}$ starting from a stable state $\mathbf{X}_m$ can be written as:

$$\mathcal{P}(\mathbf{X}|\mathbf{X}_m, t=0) = \exp(-S(\mathbf{X})) \tag{7}$$

where

$$S(\mathbf{X}) = min_{[\mathbf{X}]:\mathbf{X_m}\to\mathbf{X}} \mathcal{S}([\mathbf{x}]) \tag{8}$$

where the minimization is over all paths $[\mathbf{X}]$ starting at $\mathbf{X}_m$ and ending at $\mathbf{X}$. For $\mathbf{X}$ far away from the steady state, the probability of occupation $P(\mathbf{X})$ is equivalent to logarithmic accuracy to the probability of the most likely fluctuation, $\mathcal{P}(\mathbf{X}|\mathbf{X}_m, t=0)$ that brings the system to $\mathbf{X}$. The assumption of the form given by Eq. 7 for the occupation probability is known as the eikonal approximation.

Using a smoothness assumption for $W(\mathbf{X},\mathbf{r})$, and since the changes in numbers of A and B nodes are $O(1)$, we can neglect the difference between $W(\mathbf{X}-\mathbf{r},\mathbf{r})$ and $W(\mathbf{X},\mathbf{r})$. With this approximation, the eikonal form for the occupation probabilities in the master equation yields the following equation for $S(\mathbf{X})$ [4]:

$$H\left(\mathbf{x}, \frac{\partial s}{\partial \mathbf{x}}\right) = 0 \tag{9}$$

where

$$H(\mathbf{x},\mathbf{p}) = \sum_{\mathbf{r}} w(\mathbf{x},\mathbf{r})(\exp(\mathbf{r}\mathbf{p}) - 1) \tag{10}$$

and

$$\mathbf{x} = \mathbf{X}/N, \ \ w(\mathbf{x},\mathbf{r}) = W(\mathbf{X},\mathbf{r})/N, \ \ s(\mathbf{x}) = S(\mathbf{X})/N.$$

Eq. 9, is analogous to a Hamilton-Jacobi equation for the action of a system with Hamiltonian given by Eq. 10. The corresponding Hamilton equations of motion for components of position $\mathbf{x}$ and momentum $\mathbf{p}$ are:

$$\dot{x_i} = \frac{\partial H}{\partial p_i} \quad \dot{p_i} = -\frac{\partial H}{x_i} \tag{11}$$

with $s(\mathbf{x})$ playing the role of the classical action:

$$s([\mathbf{x}]) = \int_{[\mathbf{x}]} L(\mathbf{x}, \mathbf{p}) d\mathbf{x} = \int_{[\mathbf{x}]} \mathbf{p}\dot{\mathbf{x}} d\mathbf{x}$$

where $[\mathbf{x}]$ denotes a particular path obeying the equations of motion (Eqs. 11).

Following this Hamiltonian formulation to characterize the fluctuational paths of the system, our goal is to find the path with minimum action that reaches the separatrix in phase space of the deterministic motion, starting from the vicinity of the stable state under consideration [6, 4]. Arguments in [3] show that the fluctuational path reaching the separatrix with the minimal value of the action, is the path that passes through the saddle point. This is the *optimal escape path*, i.e., the path whose probability of occurrence dominates the probability of escape and we denote it by $[\mathbf{x}_{\rm opt}]$. This path can be found by integrating the equations of motion Eq. 11, and finding the required path that starts from the vicinity $\mathbf{x}_m$ to the saddle point $\mathbf{x}_{\rm saddle}$. Thus following Eqs. 7, 8 we have for the probability of escape from the current stable point in which the system is trapped:

$$P_{\rm escape} = P(\mathbf{x}_{\rm saddle}) \sim \exp[-Ns(\mathbf{x}_{\rm saddle})] \tag{12}$$

where

$$s(\mathbf{x}_{\rm saddle}) = \int_{[\mathbf{x}_{\rm opt}]} L(\mathbf{x}, \mathbf{p}) d\mathbf{x}$$

and the transition time (or time to escape from the steady state) follows:

$$T_{\rm switching} \sim \exp[Ns(\mathbf{x}_{\rm saddle})] \tag{13}$$

In practice we start from some point $\mathbf{x}$ in the vicinity of the stable state, and to obtain the corresponding momenta $\mathbf{p}$ and action $s(\mathbf{x})$, we employ a Gaussian approximation [4]:

$$S(\mathbf{x}) = \sum Z_{ij}(x_i - x_i^m)(x_j - x_j^m)$$

where Z satisfies an algebraic Ricatti equation:

$$\mathbf{Q}\mathbf{Z}^{-1} + \mathbf{Z}^{-1}\mathbf{Q}^T + \mathbf{K} = 0$$

where Q is the linear stability matrix (Eq. 3) evaluated at $\mathbf{x}_m$ and $K_{ij} = \sum_{\mathbf{r}} w(\mathbf{x}_m, \mathbf{r}) r_i r_j$. Solving this Ricatti equation yields Z which in turn yields $S(\mathbf{x})$ and $p(\mathbf{x})$.

In order to find the optimal fluctuational path of escape from a given steady state, we numerically generate fluctuational paths from various points close to the steady state (we explore points at intervals of 10^{-5} along the x_1 dimension and 10^{-2} along the x_2 dimension around the steady state) and find one that passes close enough (no greater than a distance of 10^{-5}) to the saddle point. The equations of motion, Eqs. 11, are integrated using a trapezoidal rule to generate these paths starting with initial conditions obtained using the Gaussian approximation described above and subsequent numerical solution of the Ricatti equation (we use a Matlab Ricatti equation solver for the latter). The scaling behavior of switching times obtained using this approach for various committed fraction values as a function of distance from second-order transition (or cusp) point are shown in Fig. 5 of the main text.

References

[1] V. I. Arnold. *Geometrical Methods in the Theory of Ordinary Differential Equations*. Springer; 2nd edition, 1988.

[2] J. Carr. *Applications of Centre Manifold Theory*. Springer; 1 edition, 1981.

[3] M. I. Dykman and M. A. Krivoglaz. Theory of fluctuational transitions between stable states of a nonlinear oscillator. *Soviet Journal of Experimental and Theoretical Physics*, 50:30, July 1979.

[4] M. I. Dykman, Eugenia Mori, John Ross, and P. M. Hunt. Large fluctuations and optimal paths in chemical kinetics. *J. Chem. Phys.*, 100(8):5735–5750, 1994.

[5] M. I. Freidlin and A. D. Wentzell. *Random Perturbations of Dynamical Systems*. Springer-Verlag, 1984.

[6] Robert S. Maier. Large fluctuations in stochastically perturbed nonlinear systems: Applications in computing. In *Applications in computing, in 1992 Lectures on Complex Systems*. Addison-Wesley, 1992.
